# The Workplace Mental Health Paradox - Why is mental ill health at work rising yet we have never spent more to prevent it?

**DOI:** 10.1192/j.eurpsy.2025.356

**Published:** 2025-08-26

**Authors:** N. Glozier, R. Morris, M. Deady, S. Harvey

**Affiliations:** 1 University of Sydney; 2 Black Dog Institute, Sydney, Australia

## Abstract

**Introduction:**

There is a prevailing paradox in workplace mental health. Never has so much been spent on prevention, intervention, and regulatory programs yet the prevalence of employee mental ill-health has not only not improved, but rates are seemingly on the increase.

**Objectives:**

Two evaluate 2 explanations (a) has the reported prevalence of specific psychosocial workplace risk and protective factors changed over the last two decades (e.g., is work getting more stressful), and (b) are there trends, and generational differences, in the impact of these factors on worsening or buffering mental health (e.g., are employees becoming less resilient).

**Methods:**

We use a 20 year population based cohort study (n=19,744).

(a) We estimated the linear trend over time (2001 to 2020), to determine the population-trends of reporting higher levels of job demands, control and complexity.

(b) To assess cohort differences in resilience to job stressors we estimated regression models predicting mental health (MHI-5 scores) by each psychosocial risk and birth cohort. Each model included the interaction between the self-reported psychosocial risk factor (independent variable) and birth-cohort (moderator variable) to estimate the dependency for each cohort. The marginal slope between the level of the risk factor and mental health for each cohort was estimated by the delta method (see below). Differences between the marginal slopes of adjacent cohorts were tested with adjustment for pairwise comparisons.

**Results:**

From 2000 to 2020 employees report trends of increased perceived job demands and decreasing autonomy in deciding how work was completed, but increasing control over when work is carried out and greater skill use(fig 1). High levels of demands have a stronger negative impact on the mental health of Millenailas than older cohorts at a similar age, and this younger cohort benefits less from the buffering effect of autonomy at work imporving mentla health (fig2).

**Image 1:**

**Figure:**
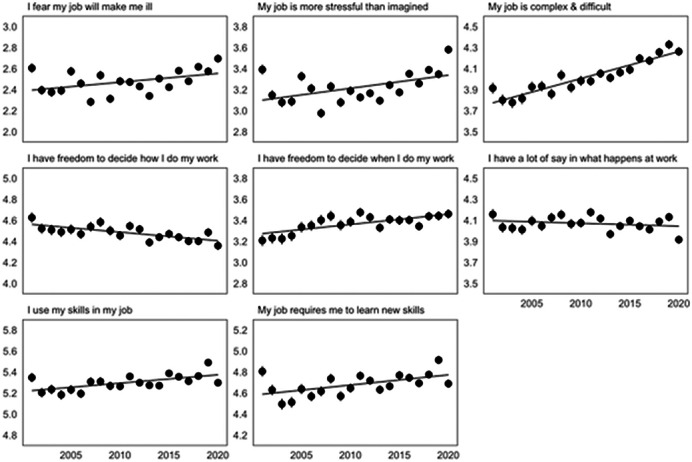

**Image 2:**

**Figure:**
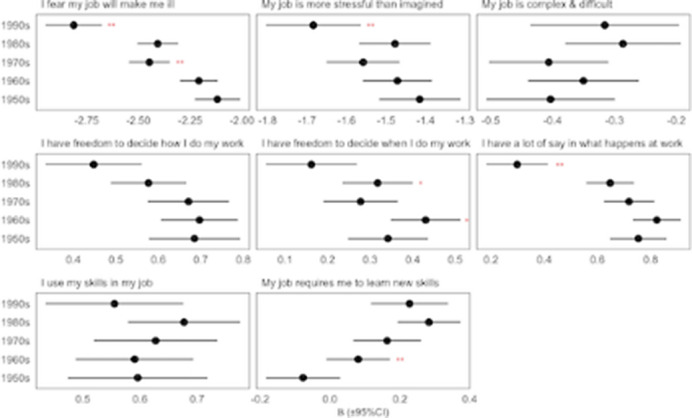

**Conclusions:**

Over the last 20 years, employees report increasing job demands and decreasing freedom in deciding how work was completed, but increasing control over when work is carried out. Although this reflects, in part, an age effect as both demands and control peak in middle age, more recent cohorts also report higher demands and less control at any given age. In addition, the marginal negative impact of higher levels of job demands on mental health has increased in younger cohorts, alongside reducing psychological benefits from autonomy and control compared with older cohorts.

This combination of employees viewing work as becoming more stressful and younger employees increased sensitivity to work stress effects may explain part of the paradox and have major implications for employers, insurers, regualtors and benefit systems.

**Disclosure of Interest:**

None Declared

